# Beyond Bioavailability: Lutein Delivery Systems and the Shift Toward Precision Nutrition

**DOI:** 10.3390/nu18213607

**Published:** 2026-10-30

**Authors:** Oluwatobi Amusat, Sun-Ok Lee

**Affiliations:** Department of Food Science, Division of Agriculture, University of Arkansas, Fayetteville, AR 72704, USA; oamusat@uark.edu

**Keywords:** lutein, carotenoids, bioavailability, encapsulation, personalized nutrition, ocular health, genetic polymorphisms

## Abstract

Lutein is a xanthophyll carotenoid and dietary pigment with an established role in ocular health and potential neuroprotective, hepatoprotective, cardiovascular, and anti-inflammatory effects. Despite this broad biological relevance, average lutein intake in Western populations remains approximately 1–2.2 mg/day, with no established RDI or RDA, while oral bioavailability is limited under typical dietary conditions. Delivery systems, including lipid-based systems, biopolymer matrices, colloidal nanocarriers, and stimuli-responsive smart platforms, have emerged as practical strategies to improve lutein stability, protect it during gastrointestinal transit, and enhance bioaccessibility and systemic exposure. However, genetic variation in uptake-related genes, including SCARB1 and CD36, alongside gut microbiome composition and body composition, may contribute to inter-individual variability in lutein response. Most existing reviews have examined lutein’s health effects, encapsulation, and delivery systems; few have addressed whether formulation performance differs meaningfully among individuals. This review integrates lutein’s chemistry, biological activity, and delivery systems, and considers how formulation design might be matched to individual absorption profiles rather than simply improving average bioavailability across a population. It distinguishes demonstrated delivery outcomes from proposed personalized applications and highlights the human evidence needed to validate this approach.

## 1. Introduction

Carotenoids are a large category of more than 1100 natural lipophilic pigments widely distributed in plants and plant-derived foods, responsible for the bright yellow, orange, and red colors of fruits and vegetables [1,2]. Plants use carotenoids in photoprotection against reactive oxygen species (ROS) [3,4,5], while animals, including humans, cannot synthesize carotenoids de novo and must obtain them from their diet [6]. Epidemiological and clinical studies have demonstrated the role of carotenoids in human health, particularly their antioxidative and anti-inflammatory activities and potential to reduce chronic disease risk [7,8].

Among carotenoids, lutein has drawn considerable scientific attention due to its antioxidant and anti-inflammatory activities and relevance to ocular and cognitive health [9]. As a xanthophyll (oxygenated) carotenoid, lutein’s structure enables it to position itself within phospholipid bilayers operating as a “molecular rivet” to protect cell membranes from oxidation as well as to increase membrane structural integrity [9,10,11].

Lutein and its structural isomer zeaxanthin are the predominant carotenoids in both the brain and the macula, and these xanthophylls make up 66–77% of the total carotenoids in the brain, but their relative proportions vary between tissues and studies [12,13]. In the retina, lutein contributes to macular pigment, filters short-wavelength blue light, and helps limit oxidative stress [14,15,16]. Beyond vision, studies have investigated its potential effects on cognitive function, hippocampal integrity, and neuroinflammation [12,17,18,19,20]. Lutein is also commercially important, used in dietary supplements, cosmetics, and food coloring [21,22].

Despite its biological importance, most people absorb very little of the lutein they eat due to lutein’s poor water solubility and susceptibility to degradation [23]. These limitations have increased interest in improving lutein stability and delivery through encapsulation and personalized approaches. The most recent advancements in encapsulation technologies such as microfluidics, nanoencapsulation (liposomes, nanoemulsions, and polymeric nanoparticles), and 3D food printing have demonstrated potential to improve lutein’s stability, controlled release, and incorporation into mixed micelles during digestion [24,25,26,27,28]. However, for these technologies to maximize lutein’s health benefits, the delivery system must account for the person receiving it.

Factors such as dietary patterns, digestive efficiency, gut microbiota composition, and genetic polymorphisms contribute to wide differences in lutein status among individuals consuming similar diets [29,30,31]. Hence, these factors must be considered when assessing whether these emerging delivery systems produce the desired health benefits. This underscores the need to tailor delivery systems to individual physiological and metabolic profiles.

Based on these developments, lutein serves as an ideal model compound to explore the connection between carotenoid chemistry, encapsulation science, and precision nutrition. Previous reviews have focused primarily on the health effects of lutein or encapsulation, while few have examined how these intersect to shape personalized delivery and precision nutrition. Thus, this review discusses the present understanding of lutein’s properties, health benefits, and bioavailability challenges, recent advances in lutein delivery systems, and the shift toward personalized nutrition, providing a framework for designing next-generation lutein delivery systems for diverse populations.

## 2. Chemistry and Biological Significance of Lutein

### 2.1. Food Sources and Consumption Patterns

Lutein cannot be synthesized *de novo* in humans [24], but is obtained via dietary intake or supplementation. Food sources include green leafy vegetables like spinach, kale, broccoli, parsley, and lettuce, as well as yellow and orange foods including maize (corn), orange peppers, carrots, and egg yolks [9,23,32,33]. Lutein and zeaxanthin concentrations vary widely among food sources, with leafy green vegetables containing approximately 0.01–40 mg/100 g [34]. Eggs contain lutein and may provide a relatively bioavailable source because of their lipid content [30,35], highlighting that not all dietary lutein is equal. Commercially, lutein is extracted from marigold flowers and is typically sold in its esterified form, which is then converted to its free form in the body [21,23].

Despite its abundance in common vegetables, human consumption remains low. Average daily intake of lutein in the US and Europe is approximately 1–2.2 mg/day, with even lower amounts reported for children [33]. No established recommended daily intake (RDI) or recommended dietary allowance (RDA) exists for lutein. Suggested intakes in the literature should be distinguished from established recommendations and clinical-trial specific supplementation doses. For example, the Age-Related Eye Disease Study (AREDS2) investigated 10 mg/day lutein together with 2 mg/day zeaxanthin in participants at risk of progression to late AMD [16,36,37].

### 2.2. Chemical Structure and Physicochemical Properties

Lutein (C40H56O2) is a xanthophyll carotenoid built on a long polyene chain containing conjugated double bonds and two cyclic end groups, each bearing a hydroxyl group [34,35]. Its characteristic yellow-orange color is due to this extensive conjugated system which acts as a chromophore, absorbing high-energy light in the blue-green spectrum (400–550 nm) [22,33].

Lutein coexists in nature with its structural isomer zeaxanthin (Figure 1), both of which are polar xanthophyll carotenoids due to the presence of two terminal hydroxyl groups on the ionone rings, which enhances their hydrophilicity compared to the non-polar carotenes [38]. The two compounds differ in the location of one double bond in the cyclic ring with lutein containing a β-ionone ring and an ε-ionone ring, whereas zeaxanthin has two β-ionone rings [39]. In nature, lutein predominantly exists in the thermodynamically stable all-trans (3R,3’R,6’R) configuration and can exist in eight possible stereoisomeric forms, arising from its three chiral centers [39,40,41].

Lutein’s distinctive structure contributes to its biological activity. The conjugated double bonds in the lutein structure allow it to donate electrons and quench singlet oxygen and reactive oxygen species (ROS) [20]. Equally important are the two terminal hydroxyl groups which allow lutein to span phospholipid bilayers and orient its polar regions toward the aqueous environment, which increases the mechanical strength and protects cells against oxidative damage [9].

#### Physicochemical Properties

Lutein (β,ϵ-carotene-3,3′-diol) is a lipophilic yellow to orange pigment with a molecular weight of 568.87 g/mol and a high melting point of approximately 183–196 °C [22]. Its conjugated double bonds are highly sensitive to oxidative degradation and isomerization when exposed to light, oxygen, and extreme pH. This poses a significant challenge for stability leading to rancid flavors, color loss, and reduced bioactivity during food processing and storage [22,39,43,44]. Lutein’s high log P (7.8) reflects its strong partitioning into lipid phases, and its low water solubility results in low bioavailability [44,45,46].

While all carotenoids are inherently lipophilic and hydrophobic, the oxygen atoms in xanthophylls like lutein render them more polar than hydrocarbon carotenes [22,33,35]. This increased polarity influences how they distribute within human tissues, as lutein is more evenly distributed among various lipoproteins in circulation compared to the purely fat-soluble carotenes [47] which has implications for how it reaches target organs.

## 3. Biosynthesis and Metabolic Fate of Lutein

### 3.1. Biosynthesis of Lutein

In photosynthetic organisms, lutein is synthesized primarily via the 2-C-methyl-D-erythritol-4-phosphate (MEP) pathway in plastids, which generate the isoprenoid building blocks dimethylallyl diphosphate (DMAPP) and isopentenyl diphosphate (IPP) from glyceraldehyde 3-phosphate and pyruvate [5]. These precursors may also be produced through an alternative cytosolic mevalonate (MVA) pathway in plants and microorganisms [48,49]. Through successive condensation, desaturation and cyclization steps, these precursors form α-carotene, which is then hydroxylated at both ring positions to form lutein [48,50,51] (Figure 2).

### 3.2. Absorption, Transport, and Tissue Distribution

The absorption of lutein is a multi-step process. Due to its lipophilic nature, lutein is emulsified into lipid droplets in the stomach and is then incorporated into mixed micelles assisted by bile salts and phospholipids in the small intestine [9,22,52,53]. Micellarization is considered the rate-limiting step for bioaccessibility, because it determines what fraction of ingested lutein is even available for absorption [54,55,56]. Lutein then enters the intestinal enterocytes primarily via facilitated transport mediated by scavenger receptor class B type 1 (SR-B1), cluster of differentiation 36 (CD36), and Niemann-Pick C1-Like 1 (NPC1L1) [30,57,58,59], though some simple diffusion can occur at high concentrations. Once inside the enterocyte, lutein is packaged into chylomicrons and secreted into the lymphatic system before entering the bloodstream [9,30,59,60].

In the systemic circulation, lutein is transported via lipoproteins by both low- density lipoproteins (LDL) and high-density lipoproteins (HDL), with HDL playing a primary role in the selective transport of lutein into the retina [9,20,35,61]. Lutein and its metabolites (3′-hydroxy-ε,ε−caroten-3-one and 3-hydroxy-β,ε-caroten-3′-one) accumulate in various tissues, including the liver, brain, skin, and adipose tissue, with the highest concentration occurring in the macula lutea [20,39,62,63]. This selective accumulation is mediated by specific binding proteins such as StARD3 (Steroidogenic Acute Regulatory Domain Protein 3) which has been identified as a primary lutein-binding protein in the human macula [29,35,64]. This tissue distribution provides a rationale for studying tissue-specific lutein effects.

## 4. Health Benefits of Lutein

Lutein’s health benefits span multiple organs. It has been investigated for ocular, neurological, hepatic, cardiovascular, and anti-inflammatory effects. Proposed mechanisms include antioxidant activity and modulation of NF-κB- and Nrf2-related signaling, although the evidence differs across tissues and experimental models (Figure 3; Table 1).

Overall, lutein’s biological activity depends on how much actually reaches the target tissue. Despite the health effects documented in Table 1, lutein’s oral bioavailability is reportedly low under typical dietary conditions [20,24], due to its hydrophobic structure, poor solubility, and susceptibility to degradation during digestion. The factors that determine how much lutein is available in the body are central to its therapeutic potential.

## 5. Lutein Bioavailability: Challenges and Factors Influencing Absorption

The therapeutic efficacy of lutein discussed above is fundamentally determined by its bioavailability, defined as the proportion of the consumed nutrient that reaches the systemic circulation and becomes available for physiological functions [22,89,90,91].

In this review, it is important to distinguish between lutein bioaccessibility (the fraction of lutein released from the food matrix and incorporated into mixed micelles), intestinal uptake (entry into enterocytes), intestinal transport (movement across the intestinal epithelium or a corresponding experimental model), systemic exposure (circulating lutein concentrations over time), tissue accumulation (lutein reaching specific organs and tissues), and biological efficacy (measurable functional outcomes at the target tissue). These are sequential but distinct processes, and improved performance at one stage does not necessarily result in improvements or greater biological effects at subsequent stages [29,53,54,55,89].

Several factors influence lutein absorption including dietary, chemical, processing, and host-related factors, and understanding these factors is important in functional food development.

### 5.1. Dietary and Food Matrix-Related Factors

The food matrix is a primary determinant of lutein’s bioaccessibility, which refers to the amount liberated from food during digestion. Lutein is often tightly bound to protein and other large molecules within plant chloroplasts, surrounded by rigid cell walls that act as physical barriers to its release [92,93]. Consequently, foods with similar lutein contents may differ substantially in bioaccessibility. For example, lutein transfer to micelles has been reported to be higher from non-green sources, such as yellow-fleshed potatoes and some fruits, than from spinach and broccoli, which have higher lutein content but lower bioaccessibility. This difference may reflect the lower chloroplast binding and greater accessibility of lutein in non-green plant tissues compared to green leafy vegetables, where lutein is tightly localized within chloroplasts and bound to pigment-protein complexes [10,22,35,94].

Dietary fat in the food matrix also plays an important role because it stimulates bile secretion, promotes micelle formation, and provides the lipid environment required for lutein solubilization and absorption [22,25,33]. It may delay gastric emptying, increasing micelle formation due to extended contact time with digestive enzymes [56]. Accordingly, lutein-enriched eggs have shown greater bioavailability than spinach or lutein supplements, likely because of the lipid content of egg yolk [95]. On the other hand, dietary fiber and divalent minerals, like calcium, may reduce absorption of lutein by interfering with micelle formation or stability [96,97,98]. These fibers increase gastrointestinal viscosity, bind bile acids which impede mixed micelle formation and affect lipase activity [56,99,100]. Pectin and alginate have also been shown to inhibit lutein absorption, likely by binding bile acids and increasing gastrointestinal viscosity [30,56,101,102].

### 5.2. Chemical Form: Free Lutein vs. Lutein Esters

The chemical form in which lutein is present in food may also influence how much is available for biological activity. However, the impact of esterification on bioavailability remains debated in the literature [21,33]. Lutein exists in nature both as free lutein and acylated with fatty acids as lutein esters [21], with the esterified form predominating in marigold-derived supplements. Esterified lutein must be hydrolyzed by intestinal esterases before absorption [34,36]. Human studies have reported conflicting results with free lutein producing a 17% greater serum response than lutein esters in a study by Norkus et al. [103]. In contrast, another study found that lutein diesters exhibited 61.6% higher bioavailability than the free form [104]. While human brush border esterases can hydrolyze lutein esters [105], the efficiency may vary with meal composition and individual digestive capacity. In practice, both forms appear broadly effective, and the choice of form for food fortification or supplementation may matter less than ensuring adequate fat co-ingestion and appropriate dosing.

### 5.3. Processing Determinants

Processing may either enhance or reduce lutein bioavailability, depending on the method used. For instance, mechanical disruptions such as chopping or pureeing disrupt cell walls, increasing the surface area for digestive enzymes which can increase the plasma response of lutein [106], while heat processing may improve lutein bioavailability compared with raw vegetables [107]. However, excessive heating can promote oxidative degradation and isomerization from the all-trans to cis configuration, which may affect biological activity [49,108]. Even so, Read et al. found that lutein bioaccessibility was higher in cookies (56.0%) and muffins (37.9%), which have higher fat contents than flatbread (23.0%), possibly because their greater fat content improved micellarization [109]. Thus, the overall effect of processing reflects a balance between cell-wall disruption, thermal degradation, and matrix composition.

### 5.4. Competition with Other Carotenoids

Competition for absorption between lutein and other carotenoids has also been identified as a significant barrier to lutein uptake [56,97]. For instance, Kostic et al. reported that β-carotene may reduce lutein absorption [110], and this inhibitory effect appears to be bidirectional as lutein also reduces β-carotene absorption, particularly when lutein is the predominant carotenoid in a meal [48,111,112]. This competition likely occurs during the transfer to mixed micelles or at shared transporter sites during chylomicron assembly [56,113]. Thus, in functional food design, carotenoid combinations need to be carefully considered as high doses of one carotenoid can affect the absorption of another.

### 5.5. Host-Related Factors: Genetics, Microbiota, and Individual Variability

Even when all dietary and processing factors are optimized, the same lutein-rich meal can produce very different plasma responses in different people. This variability may be related to genetics, the gut microbiome, and lifestyle factors including body composition.

Genetic polymorphisms account for a substantial portion of interindividual variation in lutein response. Borel et al. identified 29 single-nucleotide polymorphisms (SNPs) across 15 genes associated with postprandial lutein response in healthy men [113]. Genes associated with variability in carotenoid response include SCARB1 and CD36, which encode proteins involved directly in intestinal carotenoid uptake and transport. BCO1, which is not involved in cleavage or transport of lutein may also indirectly affect lutein absorption through downstream regulatory pathways [8,114,115,116]. However, their associations with lutein response should be interpreted according to the population and endpoint studied. SCARB1 encodes SR-B1, a key lutein transporter in intestinal cells. Genetic variants of this gene have been associated with differences in plasma carotenoid concentrations, with some estimates suggesting SCARB1 polymorphisms alone can account for substantial variation in lutein absorption [7,29]. CD36 variations at different loci have also been linked to significant differences in plasma lutein levels and macular pigment optical density (MPOD) [29,30]. BCO1 encodes an oxygenase that cleaves β-carotene into vitamin A precursors (retinoids), but its activity may indirectly affect lutein absorption. Retinoic acid induces the intestine-specific transcription factor (ISX), which represses SCARB1 and BCO1 expression. Because SR-B1 participates in lutein uptake, this pathway provides a possible indirect link between vitamin A-related signaling and lutein absorption [29,30,58,117,118].

Gut microbiota may indirectly influence lutein bioavailability by modifying bile salt profiles, bile acid excretion, intestinal transit, and colonic degradation [29,119]. However, direct evidence that gut microorganisms metabolize lutein or predict lutein absorption in humans remains limited. Differences in microbiota composition may therefore contribute to variation in lutein availability, particularly in the lower gastrointestinal tract [7,29]. Lifestyle and body composition may further affect lutein status. Diets high in ultra-processed foods have been associated with lower serum lutein levels [120] while increased adiposity may reduce circulating lutein by sequestering it in adipose tissue [20].

Together, these host-related factors suggest that lutein bioavailability depends not only on food composition but also on individual physiology. This variability provides a rationale for delivery systems that improve lutein stability and intestinal availability, although whether individual microbiota profiles alter the performance of specific lutein delivery systems remains largely unknown.

## 6. Encapsulation and Delivery Systems for Lutein

Because of lutein’s poor water solubility, sensitivity to heat, light, and oxidation, and limited uptake during gastrointestinal transit, consuming lutein-rich foods may not provide a therapeutically meaningful dose [121,122]. Encapsulation addresses these limitations by embedding lutein within a protective matrix that improves stability, solubility, and controlled release [54,123,124]. The resulting encapsulated structures form the basis of delivery systems discussed below. These systems can protect lutein during storage and digestion, mask undesirable flavors, and enhance its incorporation into mixed micelles and uptake across the intestinal epithelium [121,125,126]. For food applications, delivery systems must also be food-grade and remain stable during processing, storage, and gastrointestinal transit [127,128,129]. The main lutein delivery systems range from lipid-based carriers and biopolymer matrices, through to colloidal systems, and emerging smart platforms (Figure 4).

### 6.1. Lipid-Based Delivery Systems

Lipid-based delivery systems are among the most studied carriers for lutein because they exploit the same lipid environment that lutein naturally requires for absorption, effectively pre-packaging it in a form that already resembles a digestive intermediate. Lipid-based nanocarriers mimic physiological lipid absorption pathways, providing high biocompatibility and protection within their non-polar domains [130].

#### 6.1.1. Emulsions

These systems typically consist of lutein-loaded oil droplets dispersed in an aqueous phase, stabilized using surfactants or amphiphilic proteins [131,132]. They are preferred for lutein delivery due to the ease of preparation, scalability, and the use of natural ingredients like whey protein isolate (WPI) or soy protein isolate (SPI) as emulsifiers [133,134]. Emulsion-based systems have improved different delivery endpoints, including intestinal accumulation in animal models and plasma lutein concentrations in animal and human studies. In Wistar rat models, a soybean oil/Tween 20 emulsion produced a 15-fold increase in lutein accumulation in the lower small intestine compared with unencapsulated powder [60]. Similarly, an oleic–linoleic acid nanoemulsion increased plasma lutein concentration 2.32-fold compared to free lutein, with higher lutein accumulation also observed in liver and eye tissue in male Wistar rats [24]. In a clinical trial, a lutein nanoemulsion increased plasma lutein concentration by 1.3-fold over standard supplement capsules [135].

#### 6.1.2. Liposomes

Liposomes are spherical vesicles composed of phospholipid bilayers. Their polar and nonpolar regions allow them to accommodate different compounds, and their structural similarity to cell membranes confers high biocompatibility and protection for lutein [9,54,136,137,138]. Traditional liposome production methods may require organic solvents, which raise concern for food applications. However, supercritical carbon dioxide (SC-CO_2_) has been investigated as a solvent-free alternative, providing meaningful advantage in food applications [9,136]. Zhao et al. reported that SC-CO_2_ liposomes can achieve encapsulation efficiencies up to 97% [136].

#### 6.1.3. Solid Lipid Nanoparticles (SLNs) and Nanostructured Lipid Carriers (NLCs)

Solid Lipid Nanoparticles (SLNs) represent a first-generation solid lipid carrier composed entirely of a solid lipid matrix, while Nanostructured Lipid Carriers (NLCs) are a second-generation development that blends solid and liquid lipids to create an internal structure with greater loading capacity and improved stability [9,139,140]. Both systems can improve lutein solubility, protect against oxidative degradation, and provide controlled release [23,141].

Although SLNs may provide strong protection against UV-induced degradation, lipid crystallization can cause burst release or expulsion of the encapsulated compound. NLCs address this by mixing solid and liquid lipids, creating a less ordered internal structure that disrupts crystallinity [142,143]. In a study by Mitri et al., lutein-loaded SLNs showed only 0.06% lutein degradation compared to the 14% loss observed in liquid nanoemulsion and the 50% in corn oil suspensions, confirming that the matrix protects against UV-induced oxidation by localization of lutein in the core of the particle [144]. Omega-3-enriched NLCs have also shown high antioxidant capacity and sustained release [145].

Although SLNs can provide greater protection against UV-induced degradation than liquid emulsions [144], lipid crystallization during storage may alter release profiles and cause lutein expulsion, a limitation that does not apply to liquid emulsion systems [142,143]. Emulsions remain more amenable to large-scale food manufacturing, whereas SLN production requires tighter process control over temperature and crystallization [143].

### 6.2. Biopolymer Delivery Systems and Matrices

Biopolymer matrices based on proteins and polysaccharides provide food-compatible platforms for lutein encapsulation and may enable pH-responsive, mucoadhesive, or gastrointestinal-targeted release [54,146]. These systems use encapsulation techniques such as ionic gelation and complex coacervation to encapsulate lutein into gel-like structures (hydrogels or microspheres) through electrostatic interactions between oppositely charged polymers or ions. These mild techniques avoid high temperatures, high pressures, and toxic organic solvents, making them suitable for sensitive compounds such as lutein [54,147,148].

For instance, alginate microspheres have been designed to delay lutein release under acidic gastric conditions while promoting release at neutral intestinal pH [149]. Arunkumar et al. also reported that low molecular weight chitosan (LMWC) increased plasma lutein concentrations (systemic exposure) and tissue accumulation in the liver and eye of mice compared with bulk micelles [150]. Similarly, gelatin-octenyl succinic anhydride (OSA) microcapsules prepared via complex coacervation, improved the stability, antioxidant activity and in vitro release of lutein compared to its free form [151]. Other biopolymer delivery systems such as polysaccharide-modified liposomes, PLGA and PLGA-PEG and pH-sensitive biopolymer composites such as chitosan-alginate or zein-nanoparticles have shown potential to improve lutein stability and bioavailability [152,153,154,155,156].

Biopolymer systems offer the advantage of food-grade GRAS materials and mild processing conditions that avoid the use of organic solvents and high temperatures [54,127]. However, evidence for their performance in human studies remains limited, and whether bioaccessibility gains observed in vitro translate to improved systemic exposure and tissue accumulation in humans requires more clinical investigation [131].

### 6.3. Colloidal and Molecular Systems

These systems utilize self-assembly and molecular interactions to solubilize and protect lutein. They range from nanosized micellar assemblies to molecular-level inclusion complexes.

Micelles are formed by amphiphilic surfactants or block copolymers in an aqueous environment, creating a hydrophobic core for lutein and a hydrophilic exterior that improves water dispersibility [23,157]. The way these delivery systems are synthesized matters as kinetically controlled approaches such as electrohydrodynamic mixing-mediated nanoprecipitation (EM-NP) generally achieve better encapsulation efficiency and uniformity than thermodynamically driven methods like batch sonication. Lutein-loaded micelles from P90G lipid produced with high throughput EM-NP, achieved a 55% encapsulation efficiency and improved cellular uptake in oral epithelial cells [27,158].

Nanocrystals reduce lutein particle size via antisolvent precipitation or nanoprecipitation, increasing its surface area, and improving dissolution and oral bioavailability. In rodent models, lutein nanocrystals incorporated into oral films produced higher peak plasma concentrations than standard lutein solutions [159].

Molecular inclusion complexes physically entrap lutein within the hydrophobic cavity of a host substrate such as cyclodextrin, which protects lutein from light, oxygen and heat while substantially improving water solubility [54,160,161]. Methyl-β-cyclodextrin improved the dispersibility and bioavailability of lutein [162], while hydroxypropyl-β-cyclodextrin (HPβCD) combined with lipid nanoparticles increased corneal lutein accumulation 4.9-fold compared with lipid nanoparticles alone [163].

Cyclodextrin inclusion complexes offer notable scalability advantages, as both co-precipitation and spray drying complexation processes are industrially established [162,163,164], and β-cyclodextrin carries regulatory approval as a food additive in the EU [165]. However, improved aqueous solubility is a formulation property and does not by itself demonstrate improved bioaccessibility or guarantee equivalent improvements in intestinal uptake, systemic exposure, or tissue accumulation.

### 6.4. Emerging and Smart Delivery Systems

Newer delivery systems move beyond passive protection toward controlled release, spatial targeting, and individualized nutrient delivery.

3D food printing has emerged as a novel technology to encapsulate bioactive compounds into customized three-dimensional matrices. 3D food printing combines conventional food preparation with additive manufacturing to produce foods with complex geometric shapes and customized nutritional profiles [166,167]. The geometry and internal structure of the printed matrix directly influence lutein release kinetics, positioning 3D printing as a tool for precision nutrition [168,169,170]. Several studies have incorporated lutein into 3D-printed food matrices to improve storage stability, bioaccessibility, or intestinal transport. Coaxial 3D printing has been used to load lutein into dual-layer starch–ethyl cellulose gels, producing a 3- to 5-fold improvement in storage stability compared with unencapsulated lutein [171]. Similarly, a 3D-printed starch-zein hydrogel delivery system improved bioaccessibility and intestinal transport of lutein compared to unencapsulated lutein [172].

Microfluidics enables precise control of small fluid volumes within microchannels to fabricate delivery systems with narrow size distributions and defined core–shell structures and is now emerging as a potential method for nutrient nanoencapsulation [25,26,173,174]. Microfluidic extrusion was used to produce a noodle-like lutein food with a soy protein isolate/oil emulsion core and a calcium alginate shell. The system achieved 86% encapsulation efficiency and protected lutein under acidic gastric conditions while promoting release at neutral intestinal pH [25]. Microfluidic systems have also been used to co-encapsulate lutein and retinol in alginate nanoparticles and hydrogel beads, improving stability and antioxidant activity compared with the individual compounds [161].

Stimuli-responsive and targeted delivery systems have also been explored for lutein release under specific physiological conditions. For instance, Zhang et al. developed pH-responsive polymeric micelles that responded to the acidic pH of endosomes in human cervical cancer cells (HeLa cells), promoting lutein release at pH 5 [175]. For ocular delivery, an ion-responsive in situ gelling nanoemulsion remained liquid until contacting the ionic environment of the cornea, where it formed a gel and reduced apoptosis and reactive oxygen species (ROS) production in ARPE-19 cells [176].

Active targeting, where binding molecules are conjugated to the nanocarrier surface to exploit receptor-mediated uptake at specific cells, adds a further layer of specificity by potentially directing lutein to target tissues rather than relying on passive distribution. Biotin-conjugated PLGA–PEG nanoparticles showed higher uptake in retinal cells, suggesting potential for targeted lutein delivery to the eye. Similarly, folate-decorated nanocarriers have been investigated for lutein delivery across the blood–brain barrier, with potential relevance to neuroprotective applications in Alzheimer’s disease [9,177,178]. However, these targeted systems have been evaluated mainly in cell-based models, and their tissue specificity, safety, and efficacy require confirmation in vivo.

3D food printing and microfluidic fabrication offer opportunities to customize lutein dosage, texture, and release behavior [25,28,172], but both technologies are currently rated as low in commercial scalability, and translation from laboratory to food-grade industrial production remains a significant practical barrier [179,180]. Active-targeted nanocarriers such as biotin- and folate-decorated systems have demonstrated selective cellular uptake in cell-based models [177,178], but in vivo confirmation of their tissue specificity, safety, and efficacy for lutein delivery has not yet been reported.

Most lutein delivery systems have been evaluated primarily for physicochemical performance, including encapsulation efficiency, storage stability, solubility, and in vitro bioaccessibility (Table 2). These outcomes are important but do not establish improved biological activity. Therefore, delivery systems should also be assessed using in vivo measures of systemic exposure, tissue accumulation, and target-relevant biological outcomes. Table 3 summarizes the available in vitro and in vivo evidence for the biological performance of lutein delivery systems. Although several systems have increased plasma lutein concentrations or tissue accumulation in animal models, evidence linking encapsulation to clinically meaningful retinal, hepatic, neuronal, or cardiovascular outcomes remains limited. This gap between formulation performance and target-tissue activity is particularly important for personalized nutrition.

However, adoption of these delivery systems is not without challenges. High production costs, complex manufacturing, and limited scalability constrain which systems can realistically reach commercial food production. Further complexity may arise from the safety and regulatory pathways for new delivery systems, especially nanocarriers, adding a further layer of complexity that must be navigated before these systems can reach consumers.

### 6.5. Gut Microbiota Considerations in Lutein Delivery

Gut microbiota may influence the performance of oral lutein delivery systems, although direct evidence for lutein delivery system–microbiota interactions remains limited. Microbiota can affect bile-acid concentrations, and bile salts are essential for carotenoid micellization and intestinal bioaccessibility [7,29]. Because lipid digestion and mixed-micelle formation are critical for lutein absorption, microbiota-related differences in bile-acid metabolism may contribute to variation in the performance of lipid-based lutein carriers.

Carrier materials may also interact with the colonic environment. Polysaccharides used in oral delivery systems, including pectin, guar gum, inulin, and chitosan, act as substrates and can be degraded by colonic microbial enzymes and have therefore been investigated as microbiota-responsive materials for colon-targeted delivery [203,204]. This suggests that microbial fermentation could alter the degradation and release behavior of polysaccharide-based lutein matrices in the lower gastrointestinal tract. Conversely, stimuli-responsive systems designed to release lutein at neutral intestinal pH [25,149,161], may deliver lutein before it reaches the colon, reducing microbial exposure. However, these possibilities have not been adequately tested for most lutein delivery systems.

## 7. Safety and Regulatory Considerations

Dietary lutein is generally considered safe. A systematic risk assessment of lutein supplements used in placebo-controlled intervention trials concluded that there is strong evidence that lutein is safe at intakes up to 20 mg/day [36,38,205]. Lutein and its esters have been evaluated by the European Food Safety Authority (EFSA) and the Joint FAO/WHO Expert Committee on Food Additives (JECFA) as permissible food colorants [33]. Regulatory safety assessments apply to defined lutein preparations and intended uses, rather than to all lutein products. For example, EFSA’s 2010 evaluation established an acceptable daily intake of 1 mg/kg body weight/day for a specified lutein preparation derived from *Tagetes erecta* [206,207]. This assessment should not be treated as authorization or a universal safety limit for all encapsulated lutein formulations. The safety of each delivery system should therefore be evaluated in relation to its composition, manufacturing process, intended use, and expected exposure.

### 7.1. Carrier Materials and GRAS Status

The safety of any delivery system depends partly on the choice of carrier material. Carrier selection should consider the material’s identity, purity, intended use, exposure level, and suitability for the final food or supplement formulation [208]. Naturally derived materials such as chitosan and sodium alginate have been investigated for lutein encapsulation, but their food-use suitability and regulatory status should be verified for the specific material and intended use [135]. Synthetic polymers such as poly(lactic-co-glycolic acid) (PLGA) and polyethylene glycol (PEG) have also been used in pharmaceutical delivery systems [127,131,182]. However, their pharmaceutical use does not establish the suitability of the final lutein formulation for food use. The complete formulation, including residual solvents, impurities, and any degradation products, requires an appropriate safety assessment.

Beyond polymers, specific formulation components may also raise concerns, for instance, medium-chain triglycerides (MCTs), commonly used in nanoemulsions, were suspected at high intake quantities to negatively affect plasma lipid profiles [131,201]. Some synthetic surfactants used in emulsions have raised questions about potential intestinal barrier disruption [183]. Similarly, liposome delivery systems are vulnerable to oxidative degradation during storage, potentially generating lipid peroxidation products with cytotoxic activity, despite the phospholipids being non-toxic and highly biocompatible [127,190,191]. These concerns do not invalidate the use of these materials but emphasize the importance of formulation composition, storage conditions, and optimization even if individual components are considered safe.

### 7.2. Nano-Specific Safety Concerns

Nanoscale delivery systems require assessment of whether particles persist during digestion or dissolve or degrade before absorption. Reducing particle size to the nanoscale may change material behavior and alter their interaction with biological barriers [131,156,209]. These changes may improve lutein delivery but also raise the possibility that the carrier, rather than only lutein, may reach deeper tissues. Thus, relevant information includes particle-size distribution, composition, surface properties, stability in the food matrix, gastrointestinal degradation, exposure, and potential toxicity of the particles and their breakdown products [210]. The assessment should address the actual formulation rather than assume either safety or increased tissue penetration from particle size alone.

In vivo toxicity data for lutein-loaded nanoparticles remain limited. Acute and subacute studies of lutein-loaded PLGA nanoparticles in mice reported no adverse reactions or hematological abnormalities [209]. However, the metabolic fate of polymeric carriers and their degradation products under chronic exposure remain unclear. Toxicity concerns may be lower for micro- and macroscale systems because of their reduced ability to penetrate cell membranes, although carrier-material safety remains relevant at all scales [127].

### 7.3. Novel Food Regulation and the Path to Commercialization

The regulatory pathway for a lutein delivery system depends on its intended use, composition, manufacturing process, and jurisdiction. Conventional food ingredients, food or dietary supplements, and novel foods should not be treated as interchangeable categories. In addition, characteristics of novel delivery systems may require further safety assessment within the applicable product framework before commercialization is possible.

In the European Union, foods meeting the definition of a novel food require pre-market authorization under Regulation (EU) 2015/2283 [211]. The regulation includes engineered nanomaterials and certain foods produced using new processes [187]. Authorized uses must comply with the applicable specifications, conditions of use, and labeling requirements [187]. In the US, if the system is designed for use as a food ingredient, it should be assessed under the applicable food-additive or GRAS provisions, while dietary supplements may be subject to new dietary ingredient notification requirements [208,212]. In addition, the FDA has issued guidance on assessing how significant manufacturing changes, including nanotechnology, may affect the identity, safety, and regulatory status of food substances, but a standardized regulatory pathway for encapsulated food ingredients does not yet exist [127,187].

Therefore, the regulatory status of conventional lutein or an individual carrier component should not be assumed to cover the final encapsulated product. Commercial development requires assessment of the specific formulation and intended conditions of use. Different final formulations may exhibit distinct absorption kinetics, tissue penetration, immune interactions, and degradation behavior [127,213]. It is also worth noting that the regulatory category a product falls into (as a food supplement, food ingredient, or novel food) depends on the carrier material, particle size, and intended use, and this determines which approval pathway applies.

Consumer acceptance of novel lutein delivery formats will ultimately depend on documented product safety through rigorous toxicological evaluation and human clinical trials demonstrating safety and meaningful health benefits across diverse populations [97,214]. Thus, safety assessment should consider lutein, the carrier, particle structure, dose, and release behavior as an integrated system.

Consumer perception of novel food products presents an additional challenge that is distinct from the regulatory and safety questions. Consumer acceptance of nano-based food ingredients is lower than for conventional formulations, driven by concerns about naturalness, transparency, and unfamiliarity with the technology rather than evidence of harm [215,216]. Clear labelling, transparent communication of benefits and risks, and the use of naturally derived wall materials are likely to be important factors in gaining consumer trust for novel lutein delivery formats.

## 8. Lutein in Personalized Nutrition: From Dietary Needs to Precision Delivery

Lutein is increasingly becoming relevant in precision nutrition, because dietary intake, physiological needs, and response to supplementation vary among individuals. Although no established RDI or RDA exists for lutein, the average intake in western populations is lower than the 6–10 mg/day investigated by researchers to provide protection against conditions such as age-related macular degeneration (AMD) [22,33,36,217]. Ranard et al. assessed lutein using criteria developed by Lupton et al. for establishing dietary reference intakes and concluded that the available evidence may support DRI-like recommendations for lutein to support visual and cognitive health [33,36,218,219]. Delivery systems may help bridge the gap between current and suggested lutein intakes by supporting its incorporation into foods and suitable supplementation formats, while also improving its stability and availability for absorption.

### 8.1. Populations with Elevated Lutein Needs

Certain population groups may have increased lutein requirements or specific delivery needs that standard supplements cannot meet. Older adults are among the most important target groups. They may experience a natural decline in Macular Pigment Optical Density (MPOD) and a high prevalence of dysphagia (13–38% in independent living), which can limit their ability to swallow conventional supplement forms [16,33,54,75]. The microfluidic core–shell noodle-like system described by Yao et al. provides one example of a soft-textured lutein carrier, although it has not yet been evaluated in dysphagic adults [25].

Preterm infants represent another high-priority group. Lutein is the most abundant carotenoid in the developing brain, and preterm infants often have lower lutein concentrations than full-term infants, potentially affecting retinal and cognitive development [131,220,221,222]. However, any lutein-delivery approach for this group requires careful evaluation of dose, safety, feeding compatibility, and clinical benefit before translation to practice. In addition, individuals with high occupational screen exposure or those undergoing phototherapy may experience increased retinal oxidative stress and could benefit from delivery systems that improve lutein stability and macular availability [20,48,56,223]. However, the specific lutein requirements and clinical benefits of targeted delivery in these populations require further investigation.

### 8.2. Nutrigenomics and the Case for Personalized Dosing

Genetic variation provides a rationale for personalized lutein delivery because polymorphisms in genes involved in carotenoid absorption, transport, and metabolism have been associated with interindividual differences in postprandial lutein response [29,53,58,97]. In a randomized crossover study of 39 healthy men, Borel et al. identified 29 single-nucleotide polymorphisms across 15 genes that together explained a substantial proportion of the variability in postprandial chylomicron lutein response [58,113,118]. Variants in genes such as *SCARB1* and *CD36* may contribute to differences in intestinal uptake-related processes [29,30,58,114,115,116,117,118]. Therefore, a single lutein dose or formulation may not produce equivalent systemic or tissue exposure in all individuals [58,118].

These findings provide a rationale for testing whether individuals with lower lutein responsiveness benefit from formulations that improve lutein solubilization, mixed-micelle formation, stability during digestion, intestinal residence time, or cellular uptake. Nanoencapsulated systems may offer one approach because some carriers can enhance cellular uptake through mechanisms in addition to conventional transporter-mediated absorption as seen in Wu et al. [105,202]. However, no prospective human trials have shown that a specific delivery system can overcome genotype-associated differences in lutein absorption. Thus, genotype-informed lutein delivery remains a promising but underexplored research direction.

Genetic variation may add a second layer of complexity to the high-priority groups described in 8.1. For example, dysphagia can limit the use of conventional capsules in older adults, while genetic differences in carotenoid transport may contribute to variable absorption after intake. Soft-textured, food-based lutein formats could therefore be evaluated as practical alternatives for this population, with future studies examining whether absorption differs by genetic background. Likewise, preterm infants may have lower lutein status than full-term infants, but the influence of developmental gastrointestinal factors on lutein formulation remains unclear. Thus, future studies should determine whether life stage, delivery format, genetic variation, and gastrointestinal characteristics together predict response to specific lutein formulations.

### 8.3. The Precision Delivery Concept

Customizable and responsive delivery systems offer a potential approach to personalized lutein delivery, but their ability to improve outcomes according to individual biological profiles remains to be established.

Stimuli-responsive delivery systems that release lutein in response to pH, ionic environment, or enzymatic activity can align delivery with the physiological conditions of different gastrointestinal target sites. This is important for personalized nutrition because individuals with altered gastric pH or intestinal transit times will experience different lutein release profiles, and pH-responsive systems can in principle compensate for this variability.

3D food printing and microfluidic fabrication are rapidly developing technologies in this space, allowing for the creation of customized food matrices with precise control over lutein dosage, spatial distribution, and release kinetics [25,28,170,172,224]. These approaches could be particularly useful for individuals who require specialized food textures or dosing formats, such as older adults with dysphagia, where conventional supplement formats are not appropriate delivery vehicles [33].

High-efficiency carriers, including nanoemulsion and PLGA-based systems, have increased lutein accumulation in specific organs by up to threefold in experimental models [24,199,209,225]. These systems may offer advantages beyond increasing the oral dose. Some nanocarriers have improved cellular uptake in experimental models via endocytosis [105,131,132,226,227]. However, whether these uptake mechanisms reduce genotype-related differences in oral lutein absorption remains unknown. Altogether, these responsive delivery systems may have the potential to complement personalized nutrition strategies by aligning lutein release profiles with individual digestive conditions, absorption phenotypes, and physiological needs.

### 8.4. Research Gaps and Future Directions

Despite the progress documented in this review, several gaps remain between current understanding and the clinical implementation of personalized lutein delivery. Mechanistic studies are needed to clarify how encapsulation and delivery systems affect SR-B1-, CD36-, and NPC1L1-mediated uptake, chylomicron transport, and lymphatic uptake. Also, the influence of gut microbiome on colonic lutein metabolism, and how delivery system design interacts with microbial activity is poorly understood [59,131]. Standardized digestion models, such as INFOGEST, combined with validated cellular uptake and transport assays, could improve comparisons among delivery systems [228].

Long-term human intervention trials are particularly important. Current evidence comes largely from in vitro studies and animal models, with many studies relying on plasma lutein concentration rather than tissue-specific biological outcomes. However, there is a need for randomized trials that evaluate safety, tissue accumulation, visual or cognitive outcomes, and other clinically relevant endpoints across different age groups, health conditions, and genetic backgrounds especially for nano-scale systems [22,32,33,131]. Genotype-stratified trials are also needed to test whether personalized delivery provides greater benefits than standard supplementation.

The relationship between lutein delivery system design and gut microbiota activity represents a largely uncharacterized but potentially important dimension of lutein bioavailability that should be further investigated. Whether microbiota composition, bile-acid profiles, and microbial fermentation of carrier materials influence the release, absorption, and tissue distribution of encapsulated lutein remains poorly characterized, as discussed earlier in 6.5 and requires more mechanistic studies. Relevant experimental approaches may include standardized in vitro digestion models combined with fecal fermentation assays and in vivo pharmacokinetic studies across individuals with different microbiome profiles.

From a regulatory standpoint, novel lutein delivery systems must comply with Novel Food and related safety frameworks that are still being developed to ensure that altered kinetics of encapsulated systems do not pose pro-oxidant risks or systemic metabolic issues at high concentrations. However, its clinical value will depend on stronger mechanistic evidence, long-term human studies, and validated methods for matching formulations to individual physiological and genetic profiles.

Finally, industrial translation of advanced lutein delivery systems remains a significant practical barrier. Although many lutein delivery systems show promising laboratory-scale performance, commercial translation requires scalable manufacturing, reproducible particle characteristics, acceptable cost, and compatibility with food-processing conditions. Systems such as PLGA-based nanoparticles, microfluidic fabrications, and 3D-printed matrices, may face greater challenges related to solvent removal, batch-to-batch consistency, equipment cost, production throughput, and quality. Lipid-based systems such as nanoemulsions and SLNs may be relatively easier to scale because emulsification and homogenization are already used in food manufacturing but even these require optimization of long-term stability, cost of food-grade emulsifiers, and regulatory alignment. Therefore, future studies should report manufacturing yield, storage stability, reproducibility, processing cost, and feasibility under industrially relevant conditions, rather than laboratory performance alone.

## 9. Conclusions

Lutein is a structurally unique xanthophyll carotenoid whose conjugated double bond system and terminal hydroxyl groups contribute to its antioxidant and anti-inflammatory activities. These properties support its protective effects in tissues such as the retina and brain and may also benefit hepatic, cardiovascular, and systemic health. However, lutein intake remains low in Western populations, and its oral bioavailability is limited by poor water solubility, sensitivity to degradation, and the multiple digestive steps required for absorption.

Delivery systems and encapsulation have made real progress in addressing these limitations. Lipid-based systems, biopolymer matrices, colloidal nanocarriers, and emerging smart delivery platforms have shown formulation-specific improvements in stability, release behavior, bioaccessibility, or systemic exposure. However, whether these gains translate into measurable tissue-level benefits across diverse physiological profiles remains unclear. Thus, future research should move beyond improving bioavailability and consider interindividual differences associated with genes such as SCARB1, CD36, and BCO1. Matching delivery systems to individual absorption profiles represents a logical and tractable next step toward personalized lutein nutrition.

Long-term human trials, standardized comparison methods, and appropriate regulatory frameworks are still needed before these technologies can be widely applied. Because lutein has well-characterized tissue targets, measurable biomarkers, and biologically relevant absorption pathways, it is an ideal model for developing personalized delivery strategies for carotenoids.

## Figures and Tables

**Figure 1:**
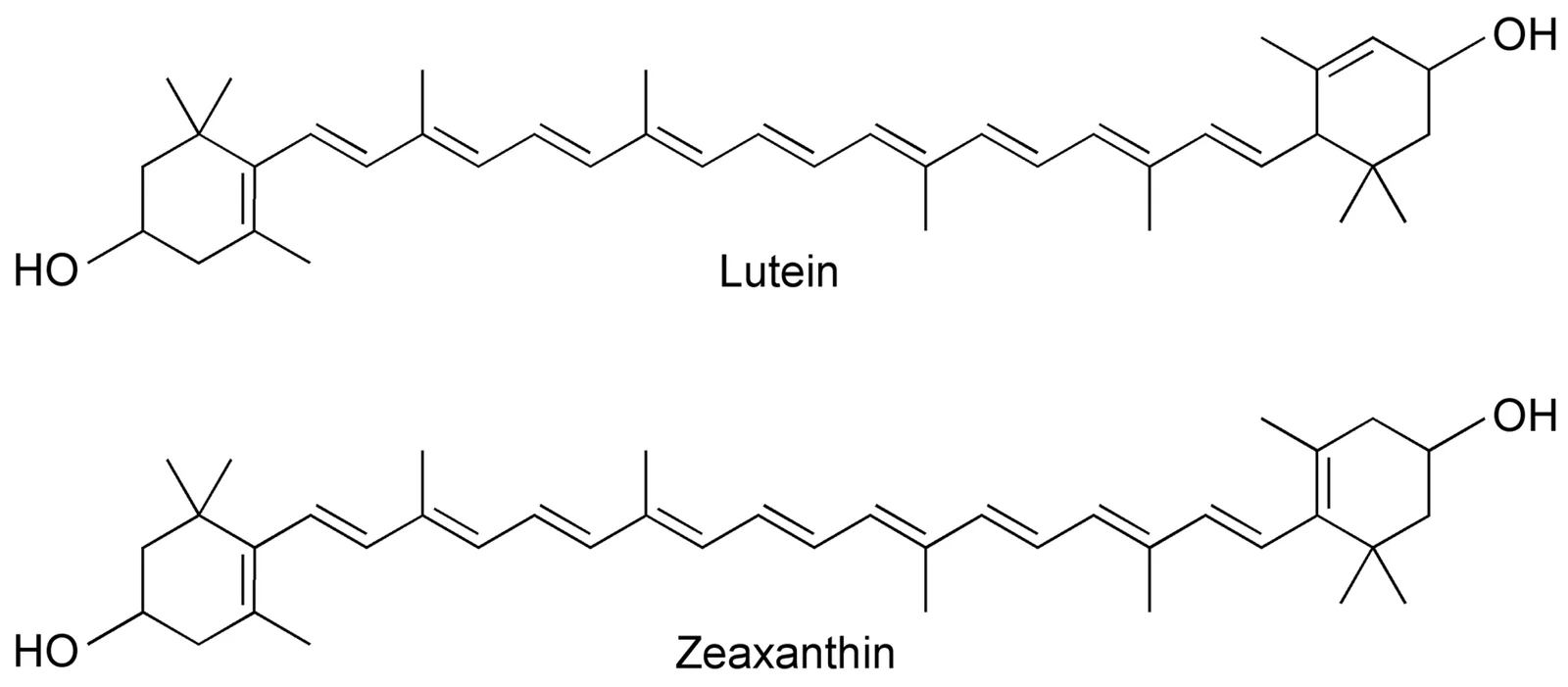
Chemical structure of lutein and zeaxanthin [42].

**Figure 2:**
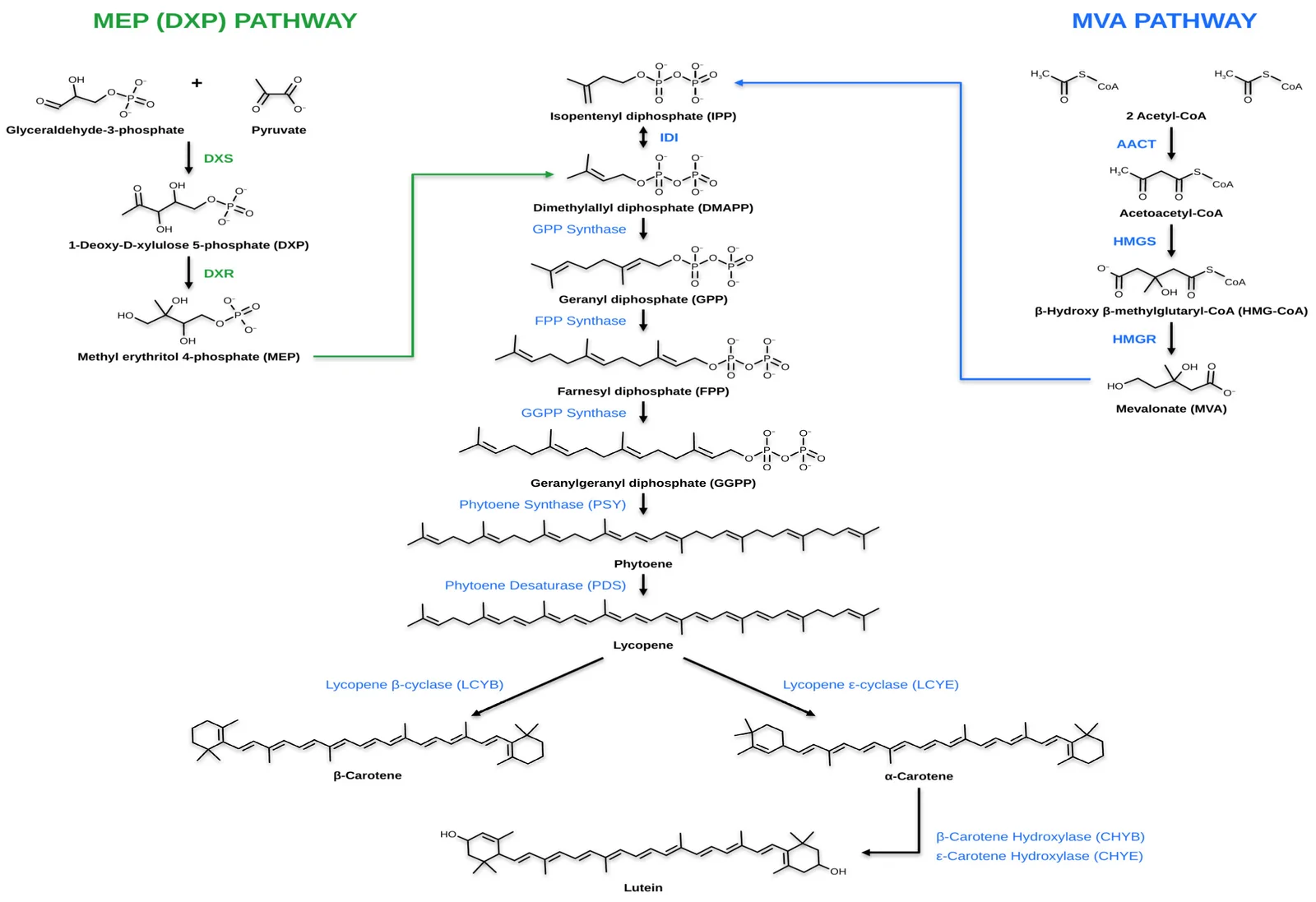
Overview of isoprenoid precursor pathways and downstream lutein biosynthesis. The MEP pathway (green) and the MVA pathway (blue) generate IPP and DMAPP in different organisms and cellular compartments. Green and blue lines indicate the entry of each pathway into the IPP/DMAPP pool. Enzymes acting downstream of IPP/DMAPP, which are shared by both routes, are also shown in blue. Black arrows indicate reaction steps; the double-headed arrow indicates reversible IPP–DMAPP isomerization. IPP: isopentenyl diphosphate; DMAPP: dimethylallyl diphosphate; GPP: geranyl diphosphate; FPP: farnesyl diphosphate; GGPP: geranylgeranyl diphosphate. Enzymes: DXS: 1-deoxy-D-xylulose 5-phosphate synthase; DXR: 1-deoxy-D-xylulose 5-phosphate reductoisomerase; AACT: acetyl-CoA C-acetyltransferase; HMGS: HMG-CoA synthase; HMGR: HMG-CoA reductase; IDI: isopentenyl-diphosphate isomerase; PSY: phytoene synthase; PDS: phytoene desaturase; LCYB: lycopene β-cyclase; LCYE: lycopene ε-cyclase; CHYB: β-carotene hydroxylase; CHYE: ε-carotene hydroxylase.

**Figure 3:**
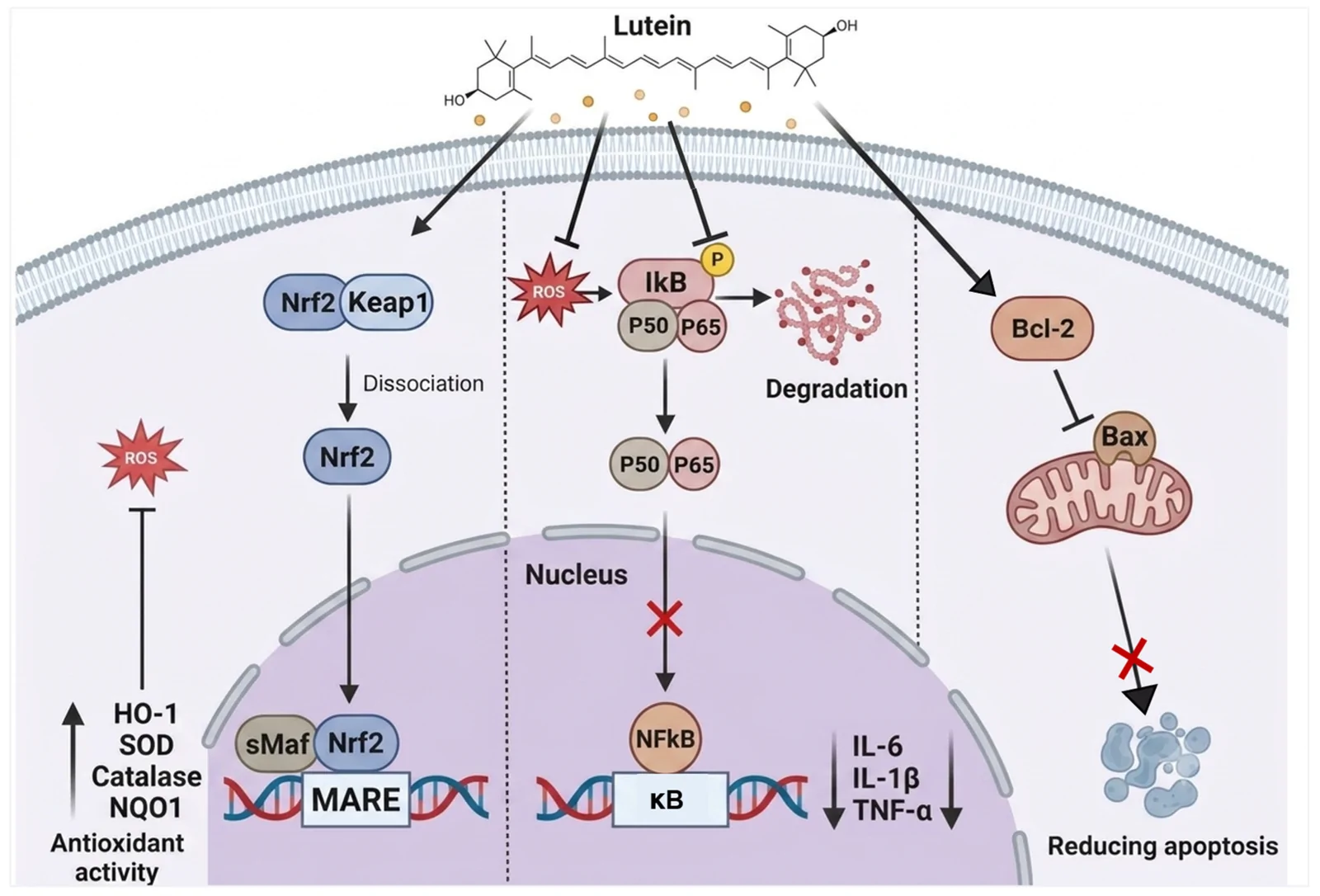
Anti-inflammatory and antioxidant mechanism of lutein. Arrows indicate activation, upregulation, or translocation; flat-ended lines indicate inhibition; red crosses indicate blocked steps. Upward and downward arrows indicate increased and decreased levels, respectively. Dotted vertical lines separate the three pathways and yellow dots represent lutein molecules. P: phosphorylation of IκB. ROS: reactive oxygen species; Nrf2: nuclear factor erythroid 2–related factor 2; Keap1: Kelch-like ECH-associated protein 1; sMaf: small musculoaponeurotic fibrosarcoma protein; ARE: antioxidant response element; HO-1: heme oxygenase-1; SOD: superoxide dismutase; NQO1: NAD(P)H quinone oxidoreductase 1; IκB: inhibitor of κB; NF-κB: nuclear factor kappa B; κB: NF-κB binding site; IL: interleukin; TNF-α: tumor necrosis factor-α; Bcl-2: B-cell lymphoma 2; Bax: Bcl-2-associated X protein.

**Figure 4:**
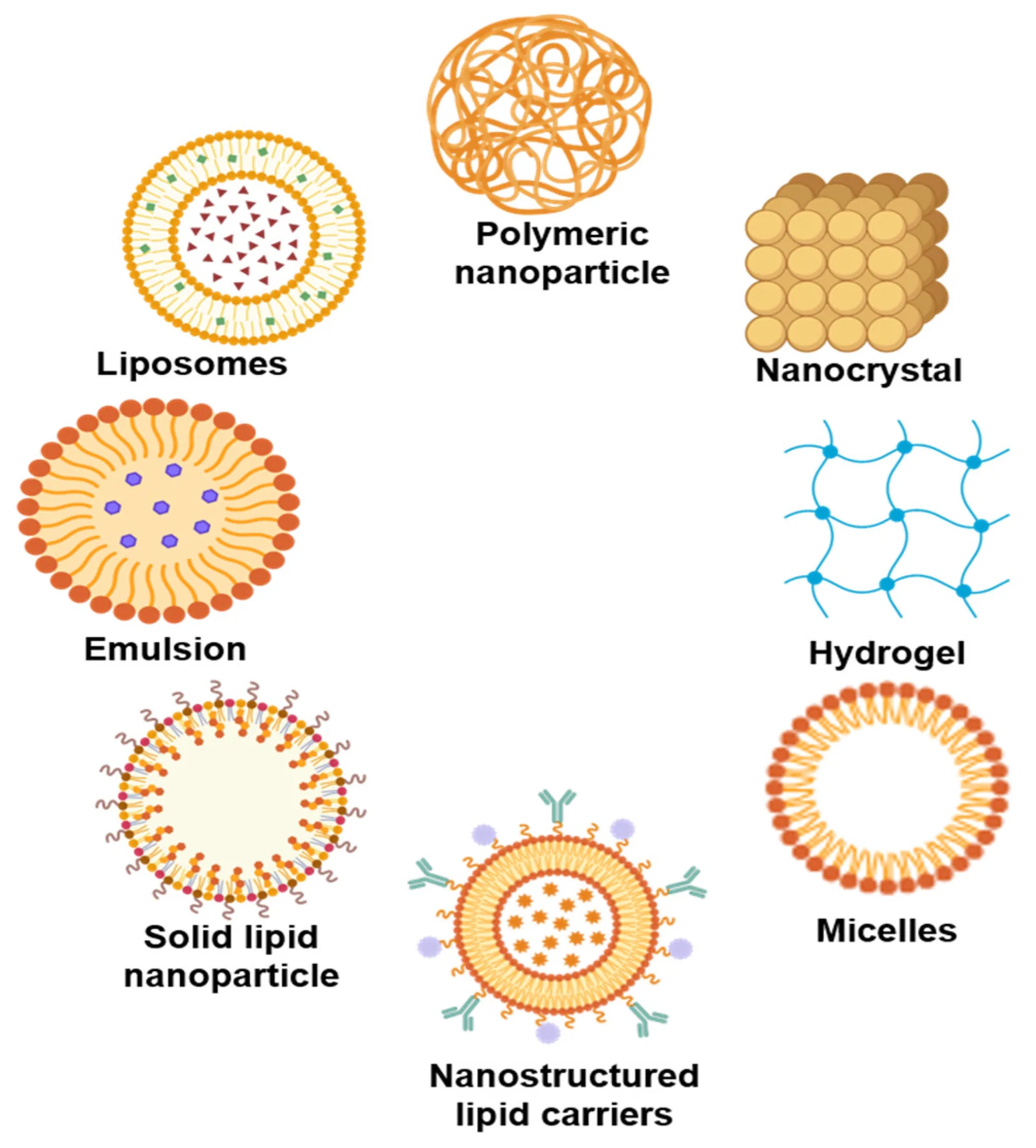
Lutein delivery systems.

**Table 1:** Summary of Lutein’s health effects.

Biological Domain	Primary Mechanism	Molecular Targets and Outcomes	Evidence	Evidence Type
Anti-inflammatory activity (Multiple tissues: Liver, lung retina, brain, macula)	▪ Blocks IκBα phosphorylation → inhibits NF-κB signaling. ▪ Activation of Nrf2/HO-1 and NQO1 → upregulates antioxidant enzymes ▪ Suppresses STAT3 activation [65]	↓ IL-1β, IL-6, TNF-α ↓ COX-2, iNOS [20,32,66] ↓ PI3K/Akt, NIK/IKK signaling [67] ↑ HO-1, NQO1 [39,65] ↓ ROS driving chronic inflammation	▪ Lutein inhibits NF-κB via H_2_O_2_ reduction [67]. ▪ 100 mg/kg reduced endotoxin-induced uveitis in animal model [68]. ▪ Additional studies have investigated reduced risk of obesity [69], diabetes [70], osteoporosis by formation of mineralized bone nodules in cultures of osteoblasts [71], gut barrier dysfunction [72,73].	▪ In vitro (RAW 264.7 cells) ▪ In vivo (animal) ▪ In vivo (Animal; Human observational)
Ocular health & Photoprotection	▪ Physical blue-light filtration (400–550 nm) before photoreceptor damage ▪ Singlet oxygen and ROS quenching ▪ NF-κB and STAT3 suppression in retinal tissue activation [65]	↑ MPOD (proxy for retinal lutein) ↑ Visual acuity ↓ IL-6 in Retina ↓ Photoreceptor apoptosis ↓ AMD progression	▪ Secondary exploratory AREDS2 analyses (10 mg/day lutein + 2 mg zeaxanthin) reduced risk of progression to advanced AMD vs. original AREDS formula containing β-carotene [74]. ▪ Supplementation studies improved MPOD, and visual acuity in AMD/maculopathy patients [62,75,76] ▪ In diabetic mice, lutein inhibited retinal ROS production and neural damage [77].	▪ In vivo (Secondary/exploratory analyses) ▪ In vivo (Supplementation trials) ▪ In vivo (Animal)
Neuroprotection and Cognitive Function	▪ NF-κB mediated neuroinflammation suppression ▪ Activation of Nrf2/HO-1/NQO1 antioxidant defenses ▪ Increased BDNF expression for neuronal survival and synaptic plasticity [78,79,80]	↓ IL-1β, IL-6, MCP-1 in brain ↑ BDNF ↓ serum ROS Positive correlation between MPOD and cognitive performance	▪ 40–160 mg/kg lutein in rats with severe traumatic brain injury (STBI), lutein significantly reduced serum ROS levels and inflammatory marker (IL-1, IL-6, and MCP-1) [81]. ▪ Preferentially accumulates in brain regions for cognition, and motor coordination [82]. ▪ Significant correlation between MPOD and cognitive function in healthy older adults [83].	▪ In vivo (Animal) ▪ In vivo (Animal) ▪ In vivo (Human studies)
Hepatoprotection	▪ NF-κB suppression via IκB-α blockade ▪ Activation of Nrf2/HO-1/NQO1 in hepatic tissue ▪ Modulates gut-liver axis → preserves intestinal barrier ▪ ↑ Lipophagy via TFEB activation	↑ Nrf2, HO-1, GST protein expression ↓ MDA (lipid peroxidation marker) ↓ Hepatic steatosis ↓ Liver injury markers ↓ Intestinal barrier dysfunction	▪ Li et al. reported that 40 mg/kg of lutein alleviated arsenic-induced hepatotoxicity via Nrf2/HO-1/GST, while reducing MDA levels and liver histological damage [84]. ▪ Lutein attenuated alcohol induced liver injury and intestinal-barrier dysfunction in rats [73]. ▪ Enhanced lipophagy and TFEB activation, associated with reduced MASLD risk [85].	▪ In vivo (Animal) ▪ In vivo (Animal) ▪ Mechanistic
Cardiovascular protection	▪ Reduces oxidative modification of LDL; ▪ Inhibits LDL- induced monocyte migration (early atherogenesis step) [20,86]. ▪ Improved endothelial-related markers; Nrf2/HO-1-in cardiac tissue [87]. ▪ NF-κB suppression	↓ Arterial wall thickening ↓Atherosclerotic lesion formation ↓ Myocardial infarction size ↓ Lipid peroxidation products ↓ CHD, stroke, metabolic syndrome risk	▪ Lutein reduced myocardial infarction size, lipid peroxidation, cardiac & apoptotic markers in a rat model, upregulated HO-1 and Nrf2 protein expression [87]. ▪ Higher blood lutein concentrations associated with lower risks of CHD, stroke, and metabolic syndrome [19]. ▪ Observational study in Beijing reported lower serum lutein levels in individuals with early atherosclerosis [88].	▪ In vivo (Animal) ▪ In vivo (Meta-analysis) ▪ In vivo (Observational study)

↑, increase or upregulation; ↓, decrease or downregulation; →, leads to or results in. Signal transducer and activator of transcription 3 (STAT3); nuclear factor kappa B (NF-κB); inhibitor of κB alpha (IκBα); interleukin (IL); tumor necrosis factor-α (TNF-α); cyclooxygenase-2 (COX-2); inducible nitric oxide synthase (iNOS); phosphoinositide 3-kinase (PI3K); protein kinase B (Akt); NF-κB-inducing kinase (NIK); IκB kinase (IKK); macular pigment optical density (MPOD); age-related macular degeneration (AMD); brain-derived neurotrophic factor (BDNF); reactive oxygen species (ROS); age-related eye disease study 2 (AREDS 2); nuclear factor erythroid 2-related factor (Nrf2); heme oxygenase-1 (HO-1); NAD(P)H Quinone Dehydrogenase 1 (NQO1); transcription Factor EB (TFEB); glutathione S-transferase (GST); malondialdehyde (MDA) metabolic dysfunction-associated steatotic liver disease (MASLD); low density lipoprotein (LDL); coronary heart disease (CHD).

**Table 2:** Comparative evaluation of lutein delivery systems.

Delivery System	Wall/Matrix Materials	Encapsulation Method	Particle Size	EE (%)	Findings—(Storage Stability, Physicochemical & Release Characteristics Stability)	Safety Considerations	Scalability	Source
Polymer Nanoparticles (PLGA-PEG)	Poly lactic-co-glycolic acid, Polyethylene glycol	Co-precipitation	80–500 nm (Mean: 200 nm)	88 ± 2	Sustained controlled-release profile (66% cumulative release over 72 h); stable colloidal suspension.	Biodegradable; pharmaceutical FDA approval does not confer food-grade status. Degradation products well-tolerated at low doses [181]. Case-by-case food regulatory evaluation required [127,182]. No adverse reactions in acute/subacute mouse studies [156].	Low—solvent-based; complex food regulatory pathway required	[156]
Polycaprolactone Nanocapsules	Polycaprolactone (PCL), MCT, Tween 80	Nanoprecipitation/Interfacial deposition	191.0–191.9 nm	99.5–99.51	No significant change in size or Z-potential over 90 days at 4 °C or 25 °C vs. initial measurements; long-term colloidal stability vs. free lutein	PCL biocompatible; approved for biomedical use only. Tween 80 at high doses may disrupt intestinal barrier [183,184]. Long-term food safety data limited.	Solvent-based; food ingredient regulatory status not established	[185]
Solid Lipid Nanoparticles (SLN)	Glyceryl monostearate, lecithin, and poloxamer-188	Ultrasonic-assisted emulsion evaporation-low temperature curing	118.50 nm	94.43	3–4 times higher thermal, light, and oxidative stability vs. free lutein	GRAS lipid matrix; well-tolerated. Poloxamer-188 is a pharmaceutical excipient—food use requires verification. Lipid crystallization may alter release profiles on storage [186].	Moderate–high—homogenization compatible with existing industrial scale-up [144].	[186]
3D-Printed Hydrogel (Starch–Ethyl Cellulose)	Corn starch (shell), ethyl cellulose (core)	Coaxial 3D food printing	0.9 mm (shell), 0.5 mm (core)	90–98	70% lutein retention after 21 days at 25 °C vs. 24% unencapsulated lutein; 5-fold improvement in retention index at 50 °C.	Wall materials are GRAS [127,171]. Regulatory requirements depend on the final formulation, manufacturing process, intended use, and jurisdiction.	Low—batch process; slow throughput; not yet food-industry scale [179]	[171]
3D-Printed Hydrogel (Zein–Corn Starch)	Zein (core), corn starch (shell)	Coaxial 3D food printing	0.9 mm (shell), 0.5 mm (core)	90–98	~61% lutein retention after 21 days at 25 °C vs. 22% unencapsulated lutein; stable under accelerated storage conditions	Zein and corn starch are GRAS. Regulatory requirements depend on the final formulation and intended use [187].	Same limitations as starch–ethyl cellulose system above [179]	[28]
Hydrogel Beads	Sodium alginate–agarose, Zein, PVP K30	Microfluidic ionotropic gelation	130 nm (NP)/0.245 cm (bead)	>84	Across different lutein: retinol loading ratios, all hydrogel formulations achieved minimal release at pH 1.2 and sustained release at pH 6.8, with improved resistance to light and thermal stress	Alginate and agarose are GRAS. PVP K30 is a pharmaceutical excipient [188] food safety data at high intake is limited. No specific lutein-loaded safety studies identified.	Low—high precision but low throughput; not food-industry scale [180].	[161]
Zein–SSPS Nanoparticles	Zein, soluble soybean polysaccharide (SSPS)	Antisolvent precipitation	200 nm	80.0	Aqueous solubility increased >30-fold vs. free lutein; excellent acid–base stability	GRAS food-derived materials. Ethanol used in precipitation must be removed to food-safe residue levels before use.	Moderate—antisolvent process scalable; ethanol removal adds a step	[189]
Nanoemulsion (Oleic–Linoleic Acid)	Oleic acid, linoleic acid, Tween-20	Sonication and magnetic stirring	110 ± 8 nm	NR	Aqueous solubility increased 726-fold; in vitro bioaccessibility 87.4% vs. 15% for free lutein	Oleic and linoleic acids are GRAS; Tween-20 approved food additive (E432, EU). Synthetic surfactants at high doses may disrupt intestinal barrier [183].	High—emulsification is industrially established and scalable	[24]
Biotin-Decorated PLGA–PEG Nanoparticles	PLGA, PEG, Biotin	Emulsion solvent evaporation	208.0 ± 3.38 nm	74.56 ± 10.25	Sustained and controlled release profile compared with free lutein; surface biotin modification enables active receptor-mediated targeting	Same PLGA/PEG safety considerations as above. Primarily a pharmaceutical platform—no food-grade regulatory pathway established.	Low—complex surface chemistry; pharmaceutical use, not suited to food-scale manufacturing	[177]
Molecular Inclusion Complex	Cyclodextrin derivatives: methyl-β-cyclodextrin (M-β-CD); hydroxypropyl-β-cyclodextrin (HPβCD)	Co-precipitation; spray drying	NR	NR	Increased lutein solubility by approximately 8-fold, 16-fold and 20- fold respectively for βCD, HP-βCD and M-βCD vs. pure lutein	Native β-CD approved as food additive (E459, EU) for specific uses HPβCD is a pharmaceutical excipient—food-grade status varies by jurisdiction [165]. High-dose native β-CD may interact with membrane cholesterol; modified derivatives preferred (HPβCD, M-β-CD).	High—industrially established; co-precipitation and spray drying amenable to large-scale manufacturing	[162,163]
Microfluidic Noodle	Alginate (shell), soy protein isolate + oil (core)	Microfluidic continuous extrusion	Shell: 0.9 mm; Core: 0.5 mm	86.0 ± 2.7	All four microfluidic noodle formulations retained >60% lutein after 7 days at 4 °C, despite a decreasing trend over storage. In vitro bioaccessibility was low (3–4%) across all formulations	All components GRAS. Soy allergen declaration required [127].	Moderate—continuous extrusion has scale-up potential; currently lab scale	[25]
Stigmasterol Liposomes	Stigmasterol, soy lecithin	Ethanol injection	221.5 ± 9.8 nm	88.23%	60% lutein retention after storage at 4 °C for 7 days; stigmasterol incorporation improves oxidative stability vs. conventional phospholipid liposome	Phospholipids and plant sterols are GRAS. Phospholipid oxidation during storage may generate cytotoxic peroxidation products [190,191]. Ethanol residues must meet food-safe limits	Moderate—ethanol injection scalable; batch-to-batch variability a challenge	[192]
Polylysine Nano-Liposomes	Polylysine (PLL), Cholesterol, Phosphatidylcholine (PC)	NR	367.1 ± 7.94 nm	92.93 ± 5.39	Sustained release in simulated gastric (51%) and intestinal (70%) fluids over 20 h; good stability against digestive fluid degradation compared to free lutein	Phosphatidylcholine and cholesterol are GRAS. Same phospholipid oxidation risk as other liposomes [190,191]	Moderate—high-pressure homogenization scalable; polylysine coating adds a step	[193]
PVA–Alginate Nanofibers	Polyvinyl alcohol (PVA), sodium alginate (SA)	NR	240–340 nm	91.9 ± 2.58	Improved hydrophilicity vs. free lutein; slow sustained release profile	Alginate is GRAS. PVA approved for food contact use; ingestion safety at high intake less established [194]	Low–moderate—electrospinning not yet food-industry scale; throughput limited	[195]
PEG Liposome–Lactoferrin (Lf-LLips)	PEG liposomes, lactoferrin	High speed shear and Ethyl hydration	186.63 ± 2.04 nm	83.11 ± 1.67	80% lutein retention after 28 days; lactoferrin surface coating improves stability compared to free lutein. Sustained in vitro release	Lactoferrin is GRAS; PEG approved food additive (E1521, EU) [196]. Phospholipid oxidation risk during storage applies [190,191]. Primarily a pharmaceutical platform—food regulatory status of the final formulation not established	Low–moderate—lactoferrin coating adds complexity; batch-to-batch consistency challenging	[197]

Note: Regulatory status depends on material identity, specifications, intended use, exposure, and jurisdiction. Ingredient-level status does not establish the safety or authorization of the complete formulation. All findings in this table reflect laboratory characterization under in vitro or bench-scale conditions. Where comparative data are reported, the comparator is stated within the findings cell. Biological performance outcomes are reported separately in Table 3. EE = encapsulation efficiency; MCT = medium-chain triglycerides; NP = nanoparticle; NR = not reported; PLGA = poly (lactic-co-glycolic acid); PEG = polyethylene glycol; SSPS = soluble soybean polysaccharide; PVA = polyvinyl alcohol; GRAS = generally recognized as safe; HPβCD = hydroxypropyl-β-cyclodextrin; SLN = solid lipid nanoparticle; SGF = simulated gastric fluid; SIF = simulated intestinal fluid.

**Table 3:** Effects of delivery systems on lutein biological performance: Evidence Across Experimental Models.

Delivery System	Experimental Model	Evidence Type	Biological Domain	Outcome Measured	Comparator	Finding	Source
Systemic exposure and oral bioavailability (Plasma)							
Chitosan Nanoparticles (LMWC)	Swiss albino mice	Animal (in vivo)	Systemic exposure	Plasma concentration 8 h after oral dose.	Mixed micelles.	54.5% higher plasma concentration	[150]
Hydrophilic Nanoemulsion	Clinical trial	Human	Systemic exposure	Plasma lutein levels after 1 week	Standard lutein supplement.	1.3-fold increase vs. standard lutein supplement.	[135]
Lutein Nanocrystals (Film)	Sprague-Dawley rats	Animal (in vivo)	Systemic exposure	Plasma Cmax and AUC	Standard lutein solution.	5.5-fold higher Cmax, and 2.1-fold higher AUC	[159]
PLGA Nanoparticles	Fischer rats	Animal (in vivo)	Systemic exposure	Relative plasma lutein availability	Free lutein.	15.6-fold higher relative availability in plasma compared to free lutein.	[155]
PLGA-PEG Nanoparticles	Swiss albino mice	Animal (in vivo)	Systemic exposure	Plasma lutein Cmax, AUC	Micellar lutein	5.4-fold higher AUC, 1.8-fold higher plasma concentration vs. micellar lutein	[156]
CHI-OA-ALG Nanoparticles	Wistar rats	Animal (in vivo)	Systemic exposure	Plasma lutein levels	Standard micellar lutein.	128.3% improvement in plasma lutein concentration after single dose exposure vs. standard micellar lutein.	[152]
Nanoemulsion (oleic-linoleic acid)	Rats	Animal (in vivo)	Systemic exposure	Peak plasma lutein concentration	Free lutein and micellar lutein	Plasma Concentration (112.6 ng/mL) vs. free lutein (48.6 ng/mL) and mixed micelles (68.5 ng/mL).	[24]
Starch-Coated Nanoparticles	Rats	Animal (in vivo)	Systemic exposure	Plasma Cmax, Tmax and AUC	Commercial reference sample (Floraglo-5% lutein)	1.9× higher Cmax and 1.4× higher AUC respectively; Tmax shortened by 1.3 h x	[198]
Tissue distribution & accumulation							
Chitosan Nanoparticles (LMWC)	Swiss albino mice	Animal (in vivo)	Ocular accumulation & hepatic accumulation	Eye tissue lutein concentration Liver concentration 8 h post dose	Mixed micelles	53.9% higher liver, and 62.8% higher eye accumulation vs. mixed micelles.	[150]
PLGA-PL Nanocarriers	Mice	Animal (in vivo)	Ocular accumulation & hepatic accumulation	Eye tissue AUC Liver AUC	Mixed micelles.	3.12-fold higher in eyes, 2.9-fold higher liver AUC vs. mixed micelles.	[199]
Lutein Ester Nanoparticles	Sprague-Dawley Rats/Kunming strain Mice	Animal (in vivo)	Tissue accumulation	Heart and kidney tissue AUC Eye tissue AUC	Bulk lutein	1.7-,1.3-,2.3-fold increase in heart, kidney, and eye AUC respectively vs. bulk lutein.	[200]
OS starch Oil-in-water Emulsion	Neonatal rats	Animal (in vivo)	Ocular & brain accumulation	Eye and brain tissue lutein	Free lutein	2.12-fold higher serum Cmax; improved lutein status in eye and brain tissues vs. free lutein	[132]
Lutein Nanoemulsion (MCT)	Guinea pigs	Animal (in vivo)	Hepatic accumulation	Hepatic lutein concentration Hepatic oxidative damage Lipid accumulation Inflammatory biomarker	Free lutein	1.5-fold higher liver lutein Reduced OxLDL vs. lutein powder and control 55% reduction in hepatic OxLDL, compared to lutein However, increases pro-inflammatory cytokine markers and cholesterol accumulation vs. free lutein	[201]
Intestinal Transport and Cellular Uptake							
Zein-Alginate-Phosphatidylcholine	Caco-2 cells	In Vitro	Intestinal transport	Basolateral secretion Cellular uptake	Standard dietary mixed micelles.	Enhanced basolateral secretion and uptake vs. standard dietary mixed micelles.	[202]
PLGA Nanoparticles	Caco-2 cells	In Vitro	Intestinal transport	Apical to basolateral secretion Cellular uptake	Micellized lutein	One-tenth the uptake vs. micellized lutein (attributed to the controlled release rate of the polymer).	[155]
3D-Printed Hydrogel (Zein–Corn Starch)	Caco-2 cells	In Vitro	Intestinal transport	Apical to basolateral secretion Cellular uptake	Lutein standard	Enhanced basolateral secretion vs. lutein standard. However, uptake was not different from lutein standard (attributed to potential transporter saturation)	[172]
Targeted Cellular Uptake and Barrier-Model Transport							
Folate-PLGA-PEG NPs	Neuroblastoma SK-N-BE cells	In Vitro	Neural-cell uptake	Intracellular lutein uptake via folate receptor endocytosis	Non decorated nanoparticles (PLGA-nanoparticles), free lutein	Enhanced uptake specifically via folate receptor-mediated endocytosis vs. non decorated nanoparticles	[178]
Biotin-Decorated NPs	Retinal cells (ARPE-19)	In Vitro	Retinal targeting	Receptor mediated cellular lutein uptake	Undecorated nanoparticles (PLGA-nanoparticles), free lutein	Increased lutein uptake by targeting specific transport systems on retinal epithelial cells vs. undecorated nanoparticles	[177]
PEG Liposome–Lactoferrin (Lf-LLips)	Blood–brain barrier (BBB) model	In Vitro	Brain targeting/BBB crossing	Permeability across BBB model	Free lutein	22.73% permeability across an in vitro BBB model	[197]
Functional biological outcomes							
PLGA-Phospholipid Nanoparticles	HepG2 cell	In Vitro	Cell viability effect	Cell viability inhibition	Free lutein at equivalent dose	Significant inhibition of HepG2cell viability vs. free lutein at equivalent dose	[199]

Note: Quantitative findings should not be directly compared across studies because doses, formulations, comparators, models, and methods differ. Increased plasma exposure or tissue accumulation does not alone establish clinical benefit. Cmax = Maximum Concentration; AUC = Area Under the concentration-time Curve; T_max_ = time to peak concentration; LMWC = Low Molecular Weight Chitosan; PLGA = Poly(lactic-co-glycolic acid); PL = Phospholipid; CHI-OA-ALG = Chitosan-Oleic Acid-Sodium Alginate, MCT= Medium chain triglyceride; PEG = polyethylene glycol; SSPS = soluble soybean polysaccharide.

## Data Availability

No new data were created or analyzed in this study. Data sharing is not applicable to this article.
